# Algorithms for detecting and analysing autocatalytic sets

**DOI:** 10.1186/s13015-015-0042-8

**Published:** 2015-04-28

**Authors:** Wim Hordijk, Joshua I Smith, Mike Steel

**Affiliations:** SmartAnalytiX.com, Lausanne, Switzerland; Biomathematics Research Centre, Department of Mathematics and Statistics, University of Canterbury, Christchurch, New Zealand

**Keywords:** Autocatalytic sets, Algorithms, Origin of life

## Abstract

**Background:**

Autocatalytic sets are considered to be fundamental to the origin of life. Prior theoretical and computational work on the existence and properties of these sets has relied on a fast algorithm for detectingself-sustaining autocatalytic sets in chemical reaction systems. Here, we introduce and apply a modified version and several extensions of the basic algorithm: (i) a modification aimed at reducing the number of calls to the computationally most expensive part of the algorithm, (ii) the application of a previously introduced extension of the basic algorithm to sample the smallest possible autocatalytic sets within a reaction network, and the application of a statistical test which provides a probable lower bound on the number of such smallest sets, (iii) the introduction and application of another extension of the basic algorithm to detect autocatalytic sets in a reaction system where molecules can also inhibit (as well as catalyse) reactions, (iv) a further, more abstract, extension of the theory behind searching for autocatalytic sets.

**Results:**

(i) The modified algorithm outperforms the original one in the number of calls to the computationally most expensive procedure, which, in some cases also leads to a significant improvement in overall running time, (ii) our statistical test provides strong support for the existence of very large numbers (even millions) of minimal autocatalytic sets in a well-studied polymer model, where these minimal sets share about half of their reactions on average, (iii) “uninhibited” autocatalytic sets can be found in reaction systems that allow inhibition, but their number and sizes depend on the level of inhibition relative to the level of catalysis.

**Conclusions:**

(i) Improvements in the overall running time when searching for autocatalytic sets can potentially be obtained by using a modified version of the algorithm, (ii) the existence of large numbers of minimal autocatalytic sets can have important consequences for the possible evolvability of autocatalytic sets, (iii) inhibition can be efficiently dealt with as long as the total number of inhibitors is small.

## Background

The concept of autocatalytic sets was introduced by Kauffman [1-3] to study the idea of life as a functionally closed and self-sustaining chemical reaction system. This concept is closely related to other such models and ideas [4-7] and is believed to have played a crucial role in the origin of life. It was later formalized mathematically in the form of RAF theory [8-10].

To briefly review RAF theory, we first define a *chemical reaction system* (CRS) as a tuple $(X,\mathcal {R},C)$ consisting of a set of molecule types *X*, a set of (possible or allowed) chemical reactions , and a catalysis set *C* indicating which molecule types can catalyse which reactions. Next, a *food setF*⊂*X* is defined as a subset of molecule types that are assumed to be freely available from the environment (i.e., they do not necessarily have to be produced by any of the reactions in ). Thus *F* is a subset of *X*, and we will denote a CRS with associated food set *F* as a quadruple $\mathcal {Q}= (X,\mathcal {R},C, F)$.

An *autocatalytic set* (or RAF set) for $\mathcal {Q}= (X,\mathcal {R},C, F)$ is a subset $\mathcal {R}' \subseteq \mathcal {R}$ of reactions which is:

1. *reflexively autocatalytic* (RA): each reaction $r \in \mathcal {R}'$ is catalysed by at least one molecule that is either present in *F* or can be formed from *F* by using a series of reactions only from $\mathcal {R}'$ itself.
2. *food-generated* (F): each reactant of each reaction in $\mathcal {R}'$ is either present in *F* or can be formed from *F* by using a series of reactions only from $\mathcal {R}'$ itself.

**Figure 1:**
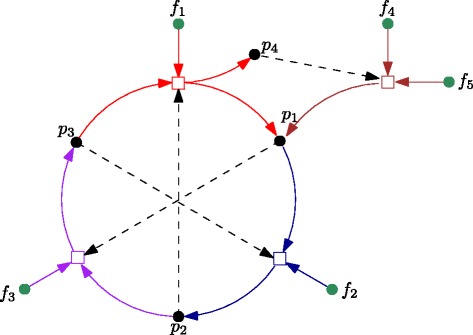
An RAF set. A simple example of an RAF set, with food set *F*={*f*_1_,*f*_2_,*f*_3_,*f*_4_,*f*_5_}. Dots represent molecule types, squares represent reactions, solid arrows indicate reactants and products, and catalysis is indicated by dashed arrows.

The first (RA) part of this definition captures the functionally closed property mentioned above; the second (F) part captures the self-sustaining property. A more formal definition of RAF sets is provided in [9,11], including an efficient (polynomial-time) algorithm for finding such sets in any (arbitrary) CRS. This RAF algorithm returns the union of all RAF (sub)sets that exist within a given CRS, or the empty set if the CRS does not contain any RAF set. Figure 1 presents a simple example of an RAF set.

As a simple model of a CRS, we use the binary polymer model as also introduced by Kauffman [2,3]. In this model, molecule types are represented by bit strings up to a certain length *n*, with the food set made up of bit strings up to a given small length *t* (e.g., *t*=2). The possible reactions are ligation (concatenating two bit strings into one larger one) and cleavage (cutting a bit string into two smaller ones). Finally, the catalysis events are assigned at random, with a given probability *p*(*x*,*r*) that a molecule *x*∈*X* catalyses a reaction $r \in \mathcal {R}$. There are several reasons to model catalysis randomly. Firstly, it is the simplest null model, and allows tractable calculations that lead to explicit formulae for the probability of RAFs, and theorems concerning their properties. Secondly, results from this simple model can be used to accurately predict the appearance of RAFs in more complex models (such as template-matching catalysis [12]). Thirdly, in general, little is known about the distribution of catalysis in real chemical systems and, as with chemical reactions, predicting catalysis is a hard problem [13] so the random model is a convenient default option. Finally, this model has also been used in other, related, computational studies on autocatalytic sets [14,15].

Using this binary polymer model, it was shown that RAF sets are highly likely to exist in general CRSs, even for very moderate and chemically plausible levels of catalysis [9,16,17]. Furthermore, this result still holds when (i) a more realistic “template-based” form of catalysis is used, where potential catalysts have to match a certain number of bits around the reaction site [11,12], (ii) only the longest polymers can act as catalysts, also in combination with the template constraint [18], (iii) a “partitioned” polymer set is used, where the polymer set is partitioned into two modules, where polymers can undergo only reactions within their own module, but catalysis can be both within and between modules, such as in an RNA/protein world [19], and (iv) when catalysis events are distributed according to a power-law distribution, resembling real-world networks [20].

The RAF sets that are found by the RAF algorithm are called *maximum* RAF sets (maxRAFs). However, it was shown that a maxRAF can often be decomposed into multiple smaller subsets which themselves are RAF sets (subRAFs) [21]. If a subRAF cannot be reduced any further without losing the RAF property, it is referred to as an *irreducible* RAF (irrRAF) set. The existence of multiple autocatalytic subsets can actually give rise to an evolutionary process [15], and the emergence of larger and larger autocatalytic sets over time [21].

Finally, RAF sets are not just a theoretical construct, but have been shown to exist in real chemical systems [22-25]. In fact, RAF theory can be applied directly and successfully to model such real chemical reaction systems [26], providing more insight into their structure and properties. Moreover, RAF sets were recently found to exist in an actual bacterial metabolic network [27].

### The basic RAF algorithm

The basic RAF algorithm [9,11] relies on the computation of the closure of the food set. Informally, the *closure*$\text {cl}_{\mathcal {R}}(Y)$ of a set of molecule types *Y* relative to a reaction set  is the set of all molecule types that can be produced by starting with *Y* and repeatedly applying reactions from  [9]. Given a CRS $(X,\mathcal {R},C,F)$, the basic RAF algorithm (as presented in [11]) is then as follows:



Equivalently, let us define a function *f* from the $2^{\mathcal {R}_{0}}$ (the set of subsets of $\mathcal {R}_{0}$) to $2^{\mathcal {R}_{0}}$ as follows: For $\mathcal {R} \subseteq \mathcal {R}_{0}$ let $f(\mathcal {R})$ be the set of reactions *r* in  that satisfy the property that all the reactants of *r* and at least one catalyst of *r* are present in $\text {cl}_{\mathcal {R}}(F)$. Using *f* we now define a sequence: $f^{k}(\mathcal {R}); k=0,1,2, \ldots $ as follows: set $f^{0}(\mathcal {R}) = \mathcal {R}$ and, for *k*≥0, define $f^{k+1}(\mathcal {R}) = f(f^{k}(\mathcal {R}))$. The algorithm then consists of recursive application of *f* to the initial set of reactions , halting at the first fixed point of *f*, which, if this set is non-empty, will be an RAF by definition. Moreover, in [9,11] it was shown that this fixed point is the maxRAF (if one exists); otherwise, the algorithm returns the empty set, in which case no RAF exists. It was also shown that the worst-case running time of this algorithm is $O(|\mathcal {R}|^{2}\log |\mathcal {R}|)$, i.e. polynomial in the size of theinput $|\mathcal {R}|$.

### Outline

In this paper, we introduce and apply a modified version of the basic RAF algorithm, which is based on the concept of a *pseudo-RAF*. This concept is related to that of a chemical organisation [28], as discussed in [29], and has useful algorithmic properties which we will explore in more detail here. We also present performance results which show that the modified algorithm is generally more efficient.

Next, we address the question of the expected (minimum) number of irreducible RAF sets that exist within an RAF set, which is an important issue for the possible evolvability of RAF sets [15]. In [21] (Theorem 1, part 1) it was proven formally that the number of irrRAFs within an RAF can grow exponentially with the size of the RAF. Since we do not know of an efficient algorithm to count the number of irrRAFs, we introduce a statistical test that provides a probable lower bound on the number of irrRAFs that can be expected to exist in any given RAF set. We apply a previously introduced extension of the RAF algorithm to randomly sample irrRAFs within an RAF to perform this statistical test.

We then introduce another extension of the basic RAF algorithm that can also handle cases where there is a small amount of inhibition, i.e., when a small number of molecules may *inhibit* certain reactions from happening. In [16], it was shown that the general problem of finding RAF sets within a CRS that includes inhibition is an NP-hard problem. However, we show here that when the number of inhibitors is limited, the problem can still be tractable, and we apply this approach in simulations.

## Pseudo-RAFs and their use in a modified RAF algorithm

In this section, we present a modified RAF algorithm which makes use of the concept of *pseudo-RAF sets*, which were briefly introduced in [29]. Here, we present a more detailed exploration of their properties, and then compare the performance of implementations of the basic and modified RAF algorithms.

### Pseudo-RAFs

Informally, a subset of reactions is a pseudo-RAF if and only if every reactant of every reaction is either a food molecule or is produced by some reaction in the set, and every reaction is catalysed by at least one molecule that is either a food molecule or is produced by some reaction in the set. For a set $\mathcal {R}'$ of reactions and a reaction $r \in \mathcal {R}$, let *ρ*(*r*) denote the set of reactants of *r*, let $\rho (\mathcal {R})$ be the union of the sets of reactants of all reactions $r \in \mathcal {R}'$; similarly, we write *π*(*r*) and $\pi (\mathcal {R})$ for the set of products of *r*, and the set of products of all reactions in $\mathcal {R}'$, respectively. The formal definition of a pseudo-RAF is then as follows:

*Definition.* Given a CRS $\mathcal {Q}=(X,\mathcal {R},C, F)$, a subset $\mathcal {R}' \subseteq \mathcal {R}$ is a *pseudo-RAF* for  if and only if for every $r \in \mathcal {R}'$ the following two conditions hold:

- $\rho (r) \subseteq F \cup \pi (\mathcal {R}')$;
- there exists $x \in F \cup \pi (\mathcal {R}')$ such that (*x*,*r*)∈*C*.

**Figure 2:**
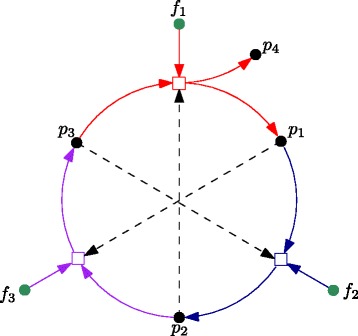
A pseudo-RAF set. A simple example of a pseudo-RAF (pRAF) with food set *F*={*f*_1_,*f*_2_,*f*_3_}. This pRAF fails to be an RAF since it is not *F*-generated (since the production of any molecule *p*_1_,*p*_2_,*p*_3_ requires both a food molecule as well as another *p*_*i*_-molecule, so this system cannot ‘get started’ just from *F*).

We will use pRAF as shorthand for pseudo-RAF. Figure 2 shows an example of a pRAF which is not an RAF.

The concept of a pRAF is related to that of a chemical organisation [28]. It is easy to show that every RAF is a pRAF, but the converse is not true, since pRAFs need not be *F*-generated (as in Figure 2). Because the *F*-generated property is necessary for a set of reactions to be capable of spontaneous generation, it is essential for an origin of life scenario. For this reason, pRAFs which are not *F*-generated are of little direct interest in this setting. However, because being a pRAF is necessary for being an RAF, and because detecting pRAFs is computationally more efficient than detecting RAFs (see below), pRAFs are algorithmically useful for detecting RAFs inside a large chemical reaction system (see below). Some basic properties of pRAFs are presented in the following lemma.

#### **Lemma1**.

Consider a CRS $\mathcal {Q}= (X, \mathcal {R}, C, F)$.

- The RAF subsets of  are precisely the *F*-generated pRAF subsets of .
- If $\mathcal {R}_{1},\mathcal {R}_{2},\dots \mathcal {R}_{k} \subseteq \mathcal {R}$ are pRAFs, then $\bigcup _{i=1}^{k}\mathcal {R}_{i}$ is a pRAF.

*Proof.* For part (i), suppose that $\mathcal {R}'$ is an RAF. Then $\mathcal {R}'$ is *F*-generated by definition, and it is also a pRAF (since every RAF satisfies the definition of a pRAF).

Conversely, suppose that $\mathcal {R}'$ is a pRAF and is *F*-generated. By Lemma 3.1 of [29], the latter is equivalent to $\text {cl}_{\mathcal {R}'}(F) = F \cup \pi (\mathcal {R}').$ Now, if we replace $F \cup \pi (\mathcal {R}')$ with $\text {cl}_{\mathcal {R}'}(F)$ in the definition of a pRAF, it follows that $\mathcal {R}'$ is an RAF.

For part (ii), by the definition of a pRAF and since

$${} \rho\left(\bigcup_{i=1}^{k} \mathcal{R}_{i} \right) = \bigcup_{i=1}^{k} \rho(\mathcal{R}_{i}) \text{ and } \pi\left(\bigcup_{i=1}^{k} \mathcal{R}_{i} \right) = \bigcup_{i=1}^{k} \pi(\mathcal{R}_{i}), $$

we have:

$$\rho\left(\bigcup_{i=1}^{k} \mathcal{R}_{i}\right) \subseteq F \cup \pi\left(\bigcup_{i=1}^{k} \mathcal{R}_{i}\right). $$

Finally, since $\pi (\mathcal {R}_{i}) \subseteq F \cup \pi \left (\bigcup _{i=1}^{k} \mathcal {R}_{i}\right)$ for 1≤*i*≤*k*, each reaction in $\bigcup _{i=1}^{k} \mathcal {R}_{i}$ is catalysed by some molecule in $F \cup \pi \left (\bigcup _{i=1}^{k} \mathcal {R}_{i}\right)$. Hence $\bigcup _{i=1}^{k} \mathcal {R}_{i}$ is a pRAF. $\hfill \square $

It follows from part (ii) that if a CRS contains a pRAF, then it contains a unique maximum pRAF, equal to the union of all the (finitely many) pRAF subsets. A similar property holds for RAF sets [9].

Finding a maximum pRAF turns out to be particularly easy and fast. This is not surprising, since the problem is formally equivalent to finding a minimal truth assignment of literals in an instance of the propositional satisfiability problem HORN-SAT, and it is well known that the latter can be solved by fast (linear-time) algorithms, such as ‘unit propagation’. This formal equivalence is described in the Appendix, but is not required further in this paper.

### A modified RAF algorithm

We now show that the basic RAF algorithm can be improved by alternating the iterations of this algorithm with the (faster) process of searching for maximum pRAFs.

Note that every application of *f* in the basic RAF algorithm requires the computation of the closure of the food set, which is computationally the most expensive part of the algorithm [9]. However, because deciding whether or not a given set of reactions is a pRAF does not require the closure computation, and because every RAF is a pRAF, the number of closure computations can be reduced by, in each iteration, first finding the maximum pRAF in the system, then checking to see if it is also an RAF. This alternating process forms the basis of our modified algorithm.

First, we define $p(\mathcal {R})$ similarly to $f(\mathcal {R})$ but replacing $\text {cl}_{\mathcal {R}}(F)$ by $F \cup \pi \,(\mathcal {R})$ in the definition. The fixed points of *p* are pRAFs. Furthermore, applying *p* recursively to  returns the maximum pRAF (if one exists) or the empty set (otherwise), in the same way that applying *f* recursively in the basic RAF algorithm returns the maxRAF or the empty set. For brevity, let $\text {fixp}(\mathcal {R})$ denote the result of applying *p* recursively to  until either a fixed point or the empty set is returned, i.e., $\text {fixp}(\mathcal {R}) = p^{n}(\mathcal {R})$ for the smallest *n*>0 such that $p^{n}(\mathcal {R}) = p^{n-1}(\mathcal {R})$ or $p^{n}(\mathcal {R}) = \emptyset $. Similarly, let $\text {fixf}(\mathcal {R})$ denote the output from applying *f* recursively (that is, $\text {fixf}(\mathcal {R})$ is the output of the previous RAF algorithm applied to ).

Recall that applying *f* requires the computation of the closure. Hence, while the basic RAF algorithm makes use of fixf, the modified algorithm avoids this computation for as long as possible. Given a CRS $Q = (X,\mathcal {R},C,F)$, the modified RAF algorithm is as follows:



This algorithm computes the largest pRAF within the current set of reactions (step 2), then checks to see if it is also an RAF, throwing out any reactions that do not conform to the RAF definition (step 4). If any reactions are thrown out, the algorithm iterates, searching for the largest pRAF within the reduced set of reactions. If at any point there are no reactions left, the algorithm stops (steps 3); otherwise, it terminates only when it discovers an RAF (step 5). In order to show that the modified algorithm terminates in exactly the same way as the basic algorithm, it remains to be shown that this RAF is guaranteed to be the maxRAF, for which we will require the monotonicity of *f* and *p*, presented in the following lemma. The final result then follows.

#### **Lemma2**.

(Monotonicity) Given a CRS $Q \,=\, (X, \mathcal {R},C, F)$, the functions $f: 2^{\mathcal {R}} \to 2^{\mathcal {R}}$ and $p: 2^{\mathcal {R}} \to 2^{\mathcal {R}}$ are monotonic.

*Proof.* Consider two subsets $\mathcal {R}_{1}, \mathcal {R}_{2} \subseteq \mathcal {R}$ such that $\mathcal {R}_{1} \subseteq \mathcal {R}_{2}$. Clearly, $\text {cl}_{\mathcal {R}_{1}}(F) \subseteq \text {cl}_{\mathcal {R}_{2}}(F)$ and hence:

(1) $${} \begin{aligned} f(\mathcal{R}_{1}) &= \{ r \in \mathcal{R}_{1} : \exists (x,r) \in C \text{ with } \rho(r) \cup \{x\} \subseteq \text{cl}_{\mathcal{R}_{1}}(F) \} \\ &\subseteq \{ r \in \mathcal{R}_{2} : \exists (x,r) \in C \text{ with } \rho(r) \cup \{x\} \subseteq \text{cl}_{\mathcal{R}_{1}}(F) \} \\ &\subseteq \{ r \in \mathcal{R}_{2} : \exists (x,r) \in C \text{ with } \rho(r) \cup \{x\} \subseteq \text{cl}_{\mathcal{R}_{2}}(F) \} \\&= f(\mathcal{R}_{2}). \end{aligned}  $$

A similar argument (replacing $\text {cl}_{\mathcal {R}_{1}}(F)$ with $F \cup \pi (\mathcal {R}_{1})$, and similarly for $\mathcal {R}_{2}$), shows that $p(\mathcal {R}_{1}) \subseteq p(\mathcal {R}_{2})$, as required. $\hfill \square $

#### **Theorem1**.

The modified RAF algorithm returns the maxRAF if  contains an RAF set; otherwise, it returns the empty set.

*Proof.* Suppose that  contains no RAF sets. Then there are no non-empty fixed points of *f*; hence the algorithm terminates only after removing all reactions, returning the empty set.

Next suppose that  contains an RAF set. It then contains a unique maxRAF set, which we will denote by $\mathcal {R}_{m}$. Clearly we have $\mathcal {R}_{m} \subseteq \mathcal {R}$. Now since $\mathcal {R}_{m}$ is a fixed point of both *f* and *p*, and by Lemma 2, we have:

$$\mathcal{R}_{m} = f(\mathcal{R}_{m}) \subseteq f(\mathcal{R}). $$

Similarly,

$$\mathcal{R}_{m} = p^{n}(\mathcal{R}_{m}) \subseteq p^{n}(\mathcal{R}) $$

for any *n*≥0, and hence $\mathcal {R}_{m} \subseteq \text {fixp}(\mathcal {R})$. Applying the same arguments recursively shows that $\mathcal {R}_{m}$ is preserved after an arbitrary number of alternating applications of fixp and *f* to .

The algorithm terminates on the first value of *k* for which $f(\mathcal {R}_{k}) = \mathcal {R}_{k}$. This terminal set of reactions $\mathcal {R}_{k}$ is therefore an RAF by definition and, by the above, we have $\mathcal {R}_{m} \subseteq \mathcal {R}_{k}$. Finally, since $\mathcal {R}_{m}$ is the maxRAF, we must have $\mathcal {R}_{m} = \mathcal {R}_{k}$, as required. $\hfill \square $

We have implemented the modified RAF algorithm; the pseudo-code of this implementation is provided in the Appendix. Briefly, to apply the modified algorithm in practice, for each molecule type it is necessary to keep track of the number of reactions in $\mathcal {R}_{k}$ that produce it (i.e. of how many reactions in $\mathcal {R}_{k}$ a given molecule type is a product). This way it will be possible to check whether a reaction in $\mathcal {R}_{k}$ still conforms to both properties of a pRAF: all of a reaction’s reactants and at least one of its catalysts need to be produced by one or more reactions in $\mathcal {R}_{k}$. If this is not the case for some reaction $r \in \mathcal {R}_{k}$, then this reaction *r* is removed in one of the pruning steps (step 2 or 4 in the modified algorithm), and the corresponding counts of the products of *r* are reduced accordingly (i.e., there is now one reaction less that produces each of *r*’s products).

In fact, in the actual implementation we only count the number of “active” reactions that produce a given molecule type. The “active” reactions are those reactions in $\mathcal {R}_{k}$ that are used at least once while computing the closure of the food set. Thus our implementation is an even stronger modification than the pRAF idea described above, although it is largely based on this idea.

In conclusion, even though we expect that the total number of calls to the closure computation procedure is reduced in the modified algorithm, its implementation does require some additional overhead (i.e., keeping track of these counts).

### Algorithm performance

**Figure 3:**
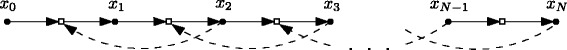
An easy CRS for the modified RAF algorithm. An example of a CRS for which the modified algorithm clearly outperforms the basic one. The original algorithm requires *N*−1 calls to the closure computation procedure, whereas the modified algorithm requires only one call. *x*_0_ is a food molecule.

A simple example shows that the modified algorithm can be substantially more efficient than the original algorithm. Consider a linear chain of reactions, as shown in Figure 3. Each reaction transforms one molecule type *x*_*i*_ into the next one *x*_*i*+1_ in the chain, and each molecule *x*_*i*_ catalyses the reaction that creates the previous molecule *x*_*i*−1_, with *x*_0_ being a food molecule. However, note that the final reaction, which creates *x*_*N*_, is not catalysed and therefore this reaction network does not form an RAF.

The basic RAF algorithm requires *N*−1 calls to the closure computation procedure to discover that there is no RAF set within this reaction network. However, the modified algorithm requires only *one* call to this procedure, as there does not exist a pRAF in this reaction network either. Applying both algorithms to an instance of this linear chain network with *N*=10,000 takes 23.3 seconds for the original algorithm and only 4.9 seconds for the modified algorithm. So, in this case, there is a clear difference in running time, a factor of 4.76. However, it is also possible to construct hypothetical examples in which the number of closure computations made by the modified algorithm, although less in absolute number, is still of the same order as in the basic algorithm. For such examples neither algorithm significantly outperforms the other.

We also compared the performance of the basic and modified algorithms (their running times and the average number of calls to the closure computation procedure) on a particular set of 100 instances of the binary polymer model with *n*=12, *t*=2, and *p*(*x*,*r*)=0.00001609 (with this value of *p*(*x*,*r*), there is about a 50% chance that an instance of the model contains an RAF set). Table 1 shows the results. As expected, the average number of calls to the closure computation procedure is reduced; indeed, by a factor of 1.78. Furthermore, the variance in this number of calls is also reduced to almost half. However, the total running time is the same between the two algorithms. When the catalysts are assigned purely randomly, as in the standard binary polymer model, the additional overhead in the modified algorithm (i.e., keeping track of the number of “active” reactions that create each reactant and catalyst in the current set) apparently cancels out the gain in speed obtained by the smaller number of closure computations, at least for this value of *n*. Also note that the average running time of the original RAF algorithm on random instances of the binary polymer model was already sub-quadratic [9], so we cannot expect too much of an improvement on the standard polymer model.

**Table 1:** **The runtime (in seconds) and average number of calls to the closure computation procedure for the two algorithms on the same set of 100 instances of the binary polymer model (with** ***n*** **=12)**

	**Runtime (sec)**	**Avg. closure calls**
		**(st.dev.)**
Basic algorithm	438	19.05
		(13.52)
Modified algorithm	439	10.69
		(7.42)

In previous work, we also applied the RAF algorithm to real reaction networks, such as a system of catalytic RNA molecules [26] and the metabolic network of *E. coli* [27]. However, these networks are too small to get useful statistics for comparing running times (which are around 30ms for these networks). Thousands or even millions of reactions are required to get useful statistics, which can easily be done with the binary polymer model, as the number of reactions grows exponentially with increasing *n*.

In short, the above results show that the modified algorithm can be expected to be not worse than the basic one in terms of running time, and better in terms of the number of calls to the closure computation procedure. Depending on the particular structure of the reaction/catalyst assignments, there can actually be a significant improvement in average running time as well.

## Sampling irreducible RAFs

An irrRAF is an RAF set for which no proper subset is an RAF set. Thus, irrRAFs represent the smallest possible RAF sets. In [21], it was shown that, in principle, there can be exponentially many irrRAFs within a maximum RAF. In general, therefore, it is not possible to enumerate all irrRAFs that exist within a given CRS efficiently. Furthermore, in [29], it was shown that even finding a *smallest* irrRAF is an NP-complete problem.

Despite their computational intractability, it would still be useful to have a better idea of the (minimum) number of irrRAFs that can be expected to exist within a given CRS. This is relevant in the context of the possible evolvability of autocatalytic sets [15,21]. In [29], a search algorithm was introduced to sample irrRAFs randomly within a given RAF set , which was subsequently used in [20] to get more insight into the size distribution of irrRAFs. Briefly, this algorithm, which is an extension of the basic RAF algorithm, works as follows (see [29] for details):



Note that the particular irrRAF that is found by this algorithm depends on the order in which the reactions in  are considered for removal. Therefore, the reactions in the given RAF  are randomly reordered (step 1) each time the algorithm is applied, so a (possibly) different irrRAF may be found each time.

It would seem that smaller irrRAFs should have a higher probability of being found by this irrRAF search algorithm than larger irrRAFs, since larger irrRAFs are more likely to be destroyed by the deletion of a (random) reaction (step 2). This bias in probability according to irrRAF size is easily seen to hold in some situations. For example, if there are just two irrRAFs, then the smaller one will be found with higher probability. If more than two irrRAFs are disjoint, this will also hold. However, it does not hold in general, as the following counterexample shows.

**Example:** Suppose a CRS with 12 reactions consists of precisely four RAFs (which are therefore irrRAFs), *A*,*B*,*C*,*D* where:

$$\begin{array}{@{}rcl@{}} A &=& \{r_{1}, r_{2}, r_{3}, r_{4}\}; B = \{r_{5}, r_{6}, r_{7}, r_{8}\}; C = \{r_{9}, r_{10}, r_{11}\}; \\D &=& \left\{r_{9}, r_{10}, r_{12}\right\}. \end{array} $$

Note that there is an overlap of two reactions (*r*_9_ and *r*_10_) in the pair of irrRAFs *C* and *D*. A careful case analysis reveals that the probabilities that the irrRAF search algorithm terminates at each one of these irrRAFs is given as follows:

$$\begin{array}{@{}rcl@{}} &&Pr[A] = Pr[B] = 0.25108225\ldots \text{and } \\&&Pr[C] = Pr[D] = 0.24891775\ldots \end{array} $$

Here, the probability of finding *any* larger irrRAF is higher than that of finding *any* smaller irrRAF, because the smaller ones have an overlap in the reactions they consist of. Therefore, the probability of finding irrRAFs of a certain size seems to depend on the amount of overlap between the various irrRAFs.

Similarly, one could ask what the probability is that the *same* irrRAF will be found more than once when applying the irrRAF search algorithm a certain number of times, especially since, in general, it cannot be known how many there are in total. However, the statistical test described next can give at least some idea of the *minimum* number of irrRAFs that can be expected to exist within an RAF set.

### A probable lower bound on the number of irrRAFs

The following is an easily applied hypothesis test on the number of irrRAFs, that has <1*%* Type 1 error. Randomly and independently apply the irrRAF search algorithm *S* times (we will assume that *S* is at least 30 or so). Now consider the following null hypothesis *H*_0_ and its alternative *H*_*a*_:

- *H*_0_: the total number of irrRAFs present in the CRS is at most *S*^2^/10.
- *H*_*a*_: the total number of irrRAFs present in the CRS is more than *S*^2^/10.

**Test:** Reject *H*_0_ in favour of *H*_*a*_ if all *S* irrRAFs returned by the search algorithm are different.

Saying this test has <1*%* Type 1 error means that if *H*_0_ were true, then one would reject *H*_0_ in favour of *H*_*a*_ no more than once in 100 times. This holds regardless of how many irrRAFs there are and how likely each one is to be found by the irrRAF search algorithm (i.e., it is independent of the amount of overlap there is between various irrRAFs). So, if sampling *S*=1000 irrRAFs with the given search algorithm found them all to be different, one would reject *H*_0_ and accept *H*_*a*_ which (in this case) says that there are at least 100,000 irrRAFs.

The justification for this test is simply by appeal to a generalisation of the well-studied “birthday problem” [30] and its Poisson approximation. In the classic birthday problem we ask what is the probability that among a sample of *S* people at least two have a birthday on the same day of the year. To solve this one focuses on the complementary event: that all the *S* people have different birthdays. Here we consider the slightly more general setting where *S* samples are drawn from *N* types (of objects) with type *i* is sampled with probability *p*_*i*_ on each draw (this specialises to the birthday problem when ‘types’ refers to ‘day of the year’, *N*=365, and *p*_*i*_ is the proportion of people born on day *i*). Then the probability *P* that *S* independent samples will comprise *S* different types is, at most, the corresponding probability *P*^′^ for the special case where *p*_*i*_=1/*N* for all *i* [30] – this is useful since the distribution of probabilities of sampling different irrRAFs no doubt varies in some complex way, but the upper bound *P*^′^ is robust to this variation. This latter probability *P*^′^ is well approximated by exp(−*λ*) for $\lambda = \binom {S}{2}/N$, and if *N*≤*S*^2^/10 we have $\lambda > 5(1-\frac {1}{N}) \approx 5$. Thus *P*≤*P*^′^≤ exp(−5)<0.01, as claimed.

We applied this hypothesis test to instances of the binary polymer model with *n*=8, *t*=2 and *p*(*x*,*r*)=0.00041 (which gives a probability *P*_*n*_≈0.5 of having an RAF set in a random model instance), using the irrRAF search algorithm described above. Taking 20 model instances that contain an RAF set, and with irrRAF samples of size *S*=10,000, all irrRAFs within each sample turn out to be different for all of these 20 instances. Even with a sample size of *S*=50,000, for most instances, all irrRAFs in the sample are different. There are some instances (less than half) where the irrRAFs in the sample are not all different, but even in those cases, there are only one or two equal pairs (out of a possible almost 2.5 billion pairs).

These results thus suggest that in most of these model instances we can expect, with 99% confidence, at least 50,000^2^/10=2.5×10^8^ irrRAFs to exist. Given that the average maxRAF size in these instances is 375 reactions, this is an astonishingly large number. It seems to indicate that having possibly exponentially many irrRAFs, as shown in [21], is not just an unlikely theoretical construct.

### The amount of overlap in irrRAF samples

Obviously, with this many irrRAFs existing within one maxRAF, there must be some overlap among them. The average irrRAF size in our model instances is about 175 reactions, slightly less than half of the average maxRAF size. Thus, they cannot all be completely disjoint. As mentioned above, this is relevant in the context of evolvability of autocatalytic sets, which requires the existence of multiple irrRAFs with a sufficient amount of variability. If all irrRAFs are mostly the same, then there is little room for different types of behavior (i.e., “attractors”). However, if the amount of similarity between irrRAFs is limited, then this can promote evolvability within the given chemical system [15,21].

To get more insight into the amount of similarity between irrRAFs we introduce two statistics: the *overlapO* and the *coverageC*, which measure (in different but related ways) the fraction of reactions in an irrRAF that are shared with other irrRAFs in the sample.

First, define the *pairwise overlapO*_*ij*_ between two irrRAFs $\mathcal {R}_{i}$ and $\mathcal {R}_{j}$ as the number of reactions they have in common, normalized by dividing by the number of reactions in the first irrRAF $\mathcal {R}_{i}$:

$$ O_{ij} = \frac{|\mathcal{R}_{i} \cap \mathcal{R}_{j}|}{|\mathcal{R}_{i}|}. $$

Thus, the pairwise overlap *O*_*ij*_ is the fraction of reactions in irrRAF $\mathcal {R}_{i}$ that are shared with irrRAF $\mathcal {R}_{j}$. If $\mathcal {R}_{i}$ and $\mathcal {R}_{j}$ are disjoint, then *O*_*ij*_=0. If $\mathcal {R}_{i}$ is a subset of $\mathcal {R}_{j}$, then *O*_*ij*_=1. Note that this measure is not symmetric, i.e., in general *O*_*ij*_≠*O*_*ji*_.

Next, the *irrRAF overlapO*_*i*_ of irrRAF $\mathcal {R}_{i}$ is the average of the pairwise overlap values *O*_*ij*_ over all irrRAFs $\mathcal {R}_{j}$ that are different from $\mathcal {R}_{i}$:

$$ O_{i} = \frac{1}{S-1} \sum_{j=1,j \neq i}^{S} O_{ij}, $$

where *S* is the total number of irrRAFs in the sample. This quantity can also be interpreted as follows. For any reaction *r* in $\mathcal {R}_{i}$ let *p*_*i*_(*r*) denote the proportion of the other *S*−1 irrRAFs that contain *r*. Then *O*_*i*_ is simply the average of these proportions across all reactions in $\mathcal {R}_{i}$. To see this, observe that

$$ p_{i}(r) =\frac{1}{S-1}\left|\left\{j \in \{1, \ldots, S\}-\{i\}: r \in \mathcal{R}_{j}\right\}\right| $$

and so the average value of *p*_*i*_(*r*) over all $r \in \mathcal {R}_{i}$ can be written as:

$$ \frac{1}{|\mathcal{R}_{i}|} \sum_{r \in \mathcal{R}_{i}} p_{i}(r) = \frac{1}{|\mathcal{R}_{i}|}\sum_{r \in \mathcal{R}_{i}} \frac{1}{S-1}\sum_{j=1, j \neq i} |\{r\} \cap \mathcal{R}_{j}|. $$

Interchanging the order of summation, and observing that $\sum _{r \in \mathcal {R}_{i}} |\{r\} \cap \mathcal {R}_{j}| = |\mathcal {R}_{i} \cap \mathcal {R}_{j}|$, we arrive at the above expression for *O*_*i*_.

It could also be useful to measure the proportion of the reactions in $\mathcal {R}_{i}$ that appear in at least one of the other *S*−1 irrRAFs. We call this the *irrRAF coverageC*_*i*_, which is defined as:

$$ C_{i} = \frac{|\bigcup_{j=1, j\neq i}^{S} (\mathcal{R}_{i} \cap \mathcal{R}_{j})|}{|\mathcal{R}_{i}|}. $$

Notice that the numerator term for *C*_*i*_ can also be written as $\left |\mathcal {R}_{i} \cap \left (\bigcup _{j=1, j \neq i}^{S} \mathcal {R}_{j}\right)\right |$, and that *C*_*i*_≤(*S*−1)*O*_*i*_, by Boole’s inequality. Comparing *O*_*i*_ and *C*_*i*_ sheds light on the pattern of intersection of $\mathcal {R}_{i}$ with the other *S*−1 irrRAFs. For example, if each of these irrRAFs intersects $\mathcal {R}_{i}$ in only a small proportion of its reactions, but collectively they contain most reactions in $\mathcal {R}_{i}$ then *O*_*i*_ will be small while *C*_*i*_ will be close to 1. On the other hand, if the other irrRAFs mostly intersect $\mathcal {R}_{i}$ in the same subset of reactions, then *O*_*i*_ and *C*_*i*_ will be similar.

Finally, the *average overlapO* is the average of *O*_*i*_ over the entire sample of irrRAFs:

$$ O = \frac{1}{S} \sum_{i=1}^{S} O_{i}. $$

The average overlap is thus the average (or expected) fraction of an irrRAF’s reactions that it shares with an (arbitrary) other irrRAF in the sample, or, equivalently, the average fraction of other irrRAFs in the sample that also contain some (arbitrary) reaction from a given irrRAF. Similary, the *average coverageC* is the average of *C*_*i*_ over the entire sample:

$$ C = \frac{1}{S} \sum_{i=1}^{S} C_{i}. $$

The average coverage is thus the average (or expected) fraction of an irrRAF’s reactions that appear in at least one other irrRAF in the sample.

**Figure 4:**
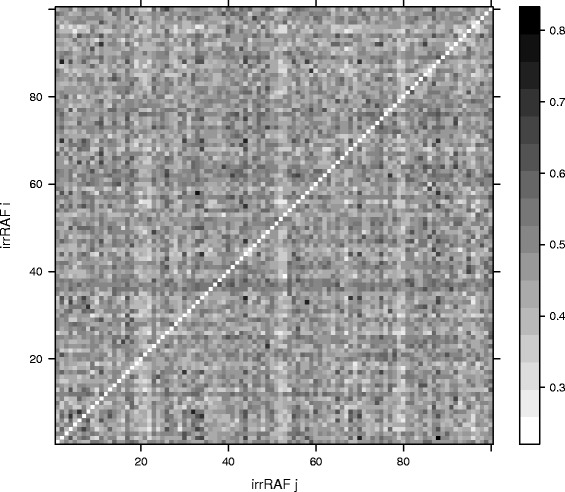
The pairwise overlap between irrRAFs. The pairwise overlap values *O*_*ij*_ for a random sample of 100 irrRAFs. The grey-scale indicates the amount of overlap (from 0 to 1) between two irrRAFs $\mathcal {R}_{i}$ and $\mathcal {R}_{j}$.

The overlap statistic is illustrated in Figure 4 for a random sample of *S*=100 irrRAFs from an instance of the binary polymer model with the same parameter values as in the previous subsection. Each cell in this grid represents the pairwise overlap *O*_*ij*_ between two irrRAFs $\mathcal {R}_{i}$ and $\mathcal {R}_{j}$ as a grey-scale value, going from white (no overlap) to black (full overlap). The minimum of all pairwise overlap values *O*_*ij*_ in this sample is 0.258 and the maximum value is 0.795. So, a given irrRAFs $\mathcal {R}_{i}$ shares anywhere between 25% and 80% of its reactions with an arbitrary other irrRAF $\mathcal {R}_{j}$. The irrRAF overlap *O*_*i*_ of irrRAF $\mathcal {R}_{i}$ is the average of the cells in row *i* (excluding the diagonal element). The average overlap *O* is the average of these row averages (or, equivalently, the average over all cells except the ones on the diagonal).

Next, we calculated the average overlap *O* and also the average coverage *C* on model instances with the same parameter values, but using a larger sample size *S*. The average overlap *O* for *S*=1,000 is *O*=0.539. So, on average, an arbitrary irrRAF $\mathcal {R}_{i}$ shares just over half of its reactions with an arbitrary other irrRAF $\mathcal {R}_{j}$ from the same sample. Equivalently, any reaction *r* from a given irrRAF $\mathcal {R}_{i}$ also appears in just over half of the other irrRAFs in the sample. The average coverage *C*, though, is almost equal to one: *C*=0.999. So, almost every reaction *r* from any given irrRAF $\mathcal {R}_{i}$ also appears in at least one other irrRAF $\mathcal {R}_{j}$ from the sample.

However, it turns out that the average overlap *O* depends partly on the sizes of the irrRAFs relative to the maxRAF they are part of. As we already know from previous studies [20,29], the average size of maxRAFs increases linearly with an increasing level of catalysis (i.e., an increasing value of *p*(*x*,*r*) for a given maximum bit string length *n*), while the average size of irrRAFs does not increase. Consequently, one would expect a smaller amount of overlap between irrRAFs for larger valuesof *p*(*x*,*r*).

**Figure 5:**
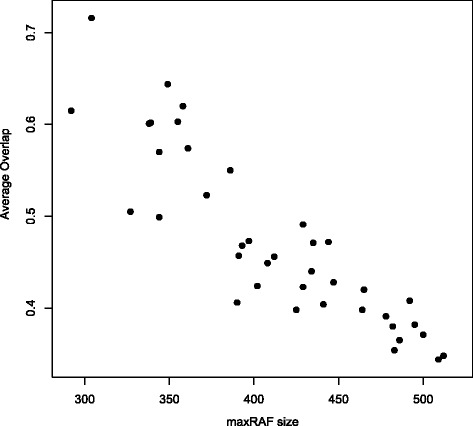
The average overlap vs maxRAF size. A scatter plot of the average overlap *O* vs. the maxRAF size for 20 binary polymer model instances with *p*=0.00041 and 20 instances with *p*=0.00045 (*n*=8 in both cases).

Indeed, calculating the average overlap *O* for instances of the binary polymer model with *p*(*x*,*r*)=0.00045 (which gives a probability *P*_*n*_≈1 of having an RAF set in a random model instance), again with *S*=1,000, gives a value of *O*=0.411, a significantly smaller value. In fact, the average overlap *O* is clearly (negatively) correlated with the size of the maxRAF, as Figure 5 shows. In this figure, the average overlap *O* vs. the maxRAF size is plotted for 20 model instances with *p*(*x*,*r*)=0.00041 and 20 instances with *p*(*x*,*r*)=0.00045 (40 points in total).

These results indicate that there is only a limited amount of overlap between pairs of irrRAFs. An arbitrary irrRAF possibly shares as little as 25% of its reactions with another irrRAF from the sample, and no more than 80%, with an average of 50% or less (depending on the value of *p*(*r*,*x*)). This implies that there is indeed sufficient variability available among irrRAFs for the potential evolvability of autocatalytic sets [15,21].

For comparison, we also calculate the exact average overlap *O* for the example of an RAF set that contains an exponential number of irrRAFs, as described in [21] (Theorem 1(1)). Recall that this example of an RAF contains *N* pairs of reactions *r*_*i*_ and *ri*′, *i*=1,…,*N*, and any irrRAF contains either *r*_*i*_ or *ri*′ for each *i*. Therefore, we have 2*N* reactions in the RAF set and there are 2^*N*^ possible irrRAFs (all of size *N*, but all being different by at least one reaction). This means that any (arbitrary) pair of irrRAFs $\mathcal {R}_{i}$ and $\mathcal {R}_{j}$ can have *k* reactions in common, where *k*=0,…,*N*−1 (if they would have *N* reactions in common, then $\mathcal {R}_{i} = \mathcal {R}_{j}$). Furthermore, there are $N \choose k$ irrRAFs $\mathcal {R}_{j}$ with which a given irrRAF $\mathcal {R}_{i}$ can have *k* reactions in common. So, by a simple combinatorial argument, the irrRAF overlap *O*_*i*_ for a given irrRAF $\mathcal {R}_{i}$ is:

$$O_{i} = \frac{1}{2^{N}-1} \sum_{k=0}^{N-1} \frac{k}{N} {N \choose k}= \frac{2^{N-1}-1}{2^{N}-1}, $$

where the second equality exploits the identity: $\sum _{k=0}^{N} k\binom {N}{k} =N2^{N-1}$. Since *O*_*i*_ is the same for each *i*=1,…,*N*, the average overlap *O* (which is the average of all values of *O*_*i*_) is also the same. Note that this quantity converges to *O*=0.5 exponentially fast with increasing *N*. For example, for *N*=3, we have *O*=0.428571…, while for *N*=10, we already have *O*=0.4995….

As a final remark on the issue of sampling irrRAFs, it would be interesting to perform the hypothesis test for the number of irrRAFs on model instances with larger values of *n*. However, the experiments described above with *n*=8 and a sample size of *S*=50,000 are already pushing current computational limits, even when using a large computer cluster. We know from previous work that the average size of maxRAFs (and also of irrRAFs) grows exponentially with increasing *n*. This means that even larger sample sizes would be required to find samples where not all irrRAFs are different. So, at present we do not expect to be able to go much beyond these limits.

## Systems that allow inhibition

The definition of a CRS is readily generalised to one which allows inhibition as well as catalysis. For example, we can prescribe a set *I* of ordered pairs (*x*,*r*), where (*x*,*r*)∈*I* means that molecule *x*∈*Xinhibits* the reaction $r \in \mathcal {R}$. Then, a CRS that allows inhibition is a tuple $(X,\mathcal {R},C,I,F)$. The definition of an RAF $\mathcal {R}'$ can then be suitably extended to require, in addition, that no reaction in $\mathcal {R}'$ is inhibited by any molecule in $\text {cl}_{\mathcal {R}'}(F)$. We refer to such an “uninhibited” RAF as a *u*-RAF.

In [16], it was shown that the problem of finding a *u*-RAF within an inhibitory CRS is an NP-hard problem. While it is possible to formulate heuristic algorithms to search for *u*-RAFs, here, we take a more precise approach that exploits some theory developed in [21].

Suppose we have a CRS $\mathcal {Q}= (X,\mathcal {R},C,I,F)$ that allows inhibition. First, we may assume, without loss of generality, that  contains no reactions that are inhibited by any element of *F* (if any such reactions exist, then we may delete them, since no such reaction can be part of any *u*-RAF). Let *X*_*I*_ denote the subset of *X* consisting of those molecules that inhibit one or more reactions. For a subset *K* of *X*_*I*_, consider the subset $\mathcal {R}_{-K}$ of  that consist of all reactions in  that do not have any product in *K* and which, in addition, are either not inhibited at all or are only inhibited by elements in *K*.

Let $\mathcal {Q}_{K}$ denote the CRS consisting of $(X, \mathcal {R}_{-K}, C, F).$

### **Proposition1**.

 has a *u*-RAF if and only if there is a subset *K* of *X*_*I*_ for which $\mathcal {Q}_{K}$ has an RAF. Moreover, each maximal *u*-RAF of  is a maxRAF of $\mathcal {Q}_{K}$ for some *K*. Thus, the number of maximal *u*-RAFs of  is at most $|\{K \subseteq X: \mathcal {Q}_{K} \text { has an RAF } \}|$.

### *Proof*.

For each *x*∈*X*_*I*_, let *R*_*x*_ denote the set of reactions in  that *x* inhibits (i.e., $R_{x}=\{r \in \mathcal {R}: (x,r) \in I\}$). Observe that $\mathcal {R}_{-K} := \mathcal {R}_{1}(K) \cap \mathcal {R}_{2}(K)$, where

$$\begin{array}{@{}rcl@{}} \mathcal{R}_{1}(K)&:=& \{r \in \mathcal{R}: \pi(r) \cap K = \emptyset\}, \text{ and } \mathcal{R}_{2}\\(K) &:=& \{r \in \mathcal{R}: r \in R_{x} \Rightarrow x \in K\}. \end{array} $$

First we establish a preliminary result:

(*) For any subset *K* of *X*_*I*_, if $\mathcal {R}' \subseteq \mathcal {R}_{-K}$ then no reaction in $\mathcal {R}'$ is inhibited by any molecule in $\text {cl}_{\mathcal {R}'}(F)-F$.

To see this, suppose this last statement did not hold (we will derive a contradiction). Then there would be some reaction *r* in $\mathcal {R}'$ which is inhibited by a molecule *x* that is produced by some reaction $r' \in \mathcal {R}'$ (i.e., *r*∈*R*_*x*_, with *x*∈*π*(*r*^′^)). Since $r \in \mathcal {R}_{2}(K)$, it follows that *x*∈*K*, and since $r' \in \mathcal {R}_{1}(K)$, it follows that *π*(*r*^′^)∩*K*=*∅*. However, this is not possible, since *x*∈*K* and *x*∈*π*(*r*^′^). This establishes (*).

Next we establish the following result:

(**) $\mathcal {R}'$ is a *u*-RAF for  if and only if $\mathcal {R}'$ is an RAF for $\mathcal {Q}_{K}$, where $K = X_{I} - \left (\bigcup _{r \in \mathcal {R}'} \pi (r)\right).$

To establish this, suppose that $\mathcal {R}' \subseteq \mathcal {R}_{-K}$ is an RAF for , for *K* as described. Then by (*), $\mathcal {R}'$ is also a *u*-RAF. Conversely, suppose that $\mathcal {R}'$ is a *u*-RAF for , and let $K = X_{I} - \left (\bigcup _{r \in \mathcal {R}'} \pi (r)\right).$ For any $r \in \mathcal {R}'$, we have *π*(*r*)∩*K*=*∅* and so $r \in \mathcal {R}_{1}(K)$, by definition of *K*. Moreover, if $r \in \mathcal {R}'$ and *r*∈*R*_*x*_, for some *x*∈*X*_*I*_, then since $\mathcal {R}'$ is a *u*-RAF, *x* cannot be an element of $\bigcup _{r \in \mathcal {R}'}\pi (r)$ and so *x*∈*K*. Thus $r\in \mathcal {R}_{2}(K)$. In summary, every $r \in \mathcal {R}'$ is an element of $\mathcal {R}_{1}(K)$ and of $\mathcal {R}_{2}(K)$, so $\mathcal {R}'$ is an RAF that forms a subset of $\mathcal {R}_{1}(K) \cap \mathcal {R}_{2}(K) =\mathcal {R}_{-K}$. This justifies (**).

We can now readily establish Proposition 1. For the first part, note that the ‘only if’ direction follows immediately from the ‘only if’ part of (**). For the ‘if’ part, if $\mathcal {R}' \subseteq \mathcal {R}_{-K}$ is an RAF for  for some subset *K* of *X*_*I*_, then $\mathcal {R}'$ is also a *u*-RAF, by (*). To establish the second part of Proposition 1, suppose $\mathcal {R}'$ is a maximal *u*-RAF for . Then, by (**), $\mathcal {R}'$ is a subset of $\mathcal {R}_{-K}$ for $K = X_{I} - \left (\bigcup _{r \in \mathcal {R}'} \pi (r)\right).$ Moreover, the maximal RAF $\mathcal {R}''$ for $\mathcal {Q}_{K}$ is also a *u*-RAF by (*), and since $\mathcal {R}''$ contains $\mathcal {R}'$, it follows by the assumption that $\mathcal {R}'$ is a maximal *u*-RAF of  that $\mathcal {R}'' =\mathcal {R}'$. The final inequality for the number of *u*-RAFs relies on the fact that if $\mathcal {Q}_{K}$ has an RAF, then $\mathcal {Q}_{K}$ contains a unique maximum RAF.

Note that our definition of a *u*-RAF implies a very strong notion of inhibition: a reaction that is (potentially) inhibited by some molecule is always excluded from being part of a *u*-RAF set if that molecule is produced by some reaction in that set. In reality, if an inhibitor is present in only very small concentrations, any reaction it might inhibit could possibly still happen at a substantial rate if a high enough concentration of its reactants (and catalysts) are present. Moreover, inhibitors do not necessarily always have a negative impact on a reaction system, as they can (and indeed do) play an important role in biological regulation.

However, given this strong notion of inhibition as a starting point, Proposition 1 provides a feasible way to determine if a CRS that allows inhibition contains a *u*-RAF and, if so, to find maximal ones, provided that *X*_*I*_ is small (by applying the RAF algorithm across all the subsets of *X*_*I*_).

### Application to the binary polymer model

We have implemented an extension of the RAF algorithm to search for *u*-RAFs as follows. Given a CRS $\mathcal {Q}=(X,\mathcal {R},C,I,F)$ that allows inhibition, for each of the $\phantom {\dot {i}\!}2^{|X_{I}|}$ possible subsets *K*⊆*X*_*I*_ we construct $\mathcal {R}_{-K}$ by removing all reactions from  that either:

1. have a product that is in the subset *K*, or
2. are inhibited by one or more molecule types that are not in the subset *K*.

We then apply the usual RAF algorithm to $\mathcal {Q}_{K}=(X,\mathcal {R}_{-K},C,F)$. Each time the RAF algorithm returns a non-empty set $\mathcal {R}'$, this is taken as a *u*-RAF of .

To apply this algorithm, we use an extension of the binary polymer model that also includes inhibition. After constructing an instance $\mathcal {Q}=(X,\mathcal {R},C,F)$ of the “standard” model (using the parameters *n*, *t* and *p*(*x*,*r*)), we include inhibition as follows:

1. Choose a random subset *X*_*I*_ of size *m* from *X*−*F* (i.e., no food molecules can be inhibitors).
2. For each pair (*x*,*r*) with *x*∈*X*_*I*_ and *r*∈*R*, assign (*x*,*r*) to *I* (the inhibition set) with some (identical and independent) probability *q*(*x*,*r*).

Using the parameter values *n*=10, *t*=2, *p*(*x*,*r*)=0.0000792 (giving a probability of about 0.5 of finding regular RAFs), and *m*=10 (i.e., 10 inhibitors), we applied the *u*-RAF algorithm to several instances of this extended binary polymer model allowing inhibition. Note that in this case, there are 2^10^=1024 possible subsets *K*, which means we need to apply the “regular” RAF algorithm that many times on each instance.

The results strongly depend on the value for the parameter *q*(*x*,*r*) (the probability that an inhibitor actually inhibits an arbitrary reaction). Clearly, if *q*(*x*,*r*) is too low, then almost every (regular) RAF $\mathcal {R}'$ is also a *u*-RAF. The probability that a (random) inhibitor (of which there are only *m*=10) is in the closure $\text {cl}_{\mathcal {R}'}(F)$*and* also inhibits one or more reactions in the (regular) maxRAF $\mathcal {R}'$ is simply too small. Indeed, when *q*(*x*,*r*)=*p*(*x*,*r*), the largest *u*-RAF found (among the 1024 possible ones) is always the same size as the (regular) maxRAF. Since we have only removed reactions from  to find *u*-RAFs and have not added any, this means that this largest *u*-RAF must be the same as the maxRAF (*c.f.* Proposition 1 (iii)).

However, when *q*(*x*,*r*) is larger, inhibitors do have an impact on the RAF sets. For example, using *q*(*x*,*r*)=10×*p*(*x*,*r*), the average maxRAF size is 1428 reactions (averaged over 10 model instances), while the largest *u*-RAF found is, on average, 1417 reactions (i.e., 11 reactions less than the maxRAF). With *q*(*x*,*r*)=100×*p*(*x*,*r*), the average maxRAF size is 1439, while the largest *u*-RAF is of size 1378 (i.e., 61 reactions less than the maxRAF, again averaged over 10 model instances). In fact, on two additional instances (not included in the above average), there was a maxRAF but no *u*-RAFs; none of the 1024 possible subsets $\mathcal {R}_{-K}$ contained an RAF set.

**Figure 6:**
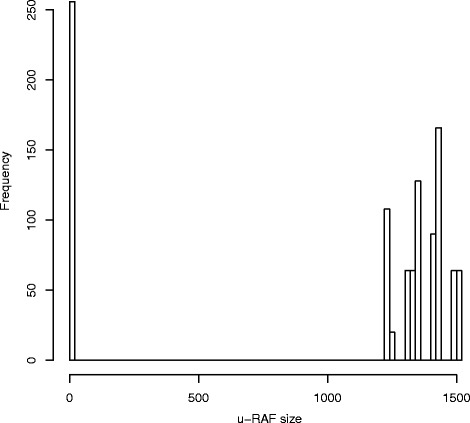
The size distribution of *u*-RAFs. A histogram of the *u*-RAF sizes for one particular instance of the binary polymer model that allows inhibition. The largest *u*-RAF is of size 1508, while the “regular” maxRAF of this instance is of size 1525.

Note that there is not necessarily just one maximal *u*-RAF (as opposed to always having only one “regular” maxRAF). In fact, *u*-RAFs of different sizes are found within the set of 2^*m*^ possible *u*-RAFs. Figure 6 shows a histogram of the 2^10^=1024*u*-RAF sizes obtained for one particular model instance with *q*(*x*,*r*)=10×*p*(*x*,*r*). In this case, 256 subsets *K*⊂*X*_*I*_ result in an empty RAF (and therefore no *u*-RAF), while for the remaining 768 subsets *K*, the *u*-RAF sizes range from 1233 to 1508, whereas the size of the “regular” maxRAFis 1525.

A full investigation of the impact of inhibition on RAF sets is beyond the scope of the current paper. Our purpose here is to introduce the notion of *u*-RAFs and an extended RAF algorithm for finding them, and to show that an actual implementation of this *u*-RAF algorithm can be successfully applied to reaction networks allowing inhibition. A more detailed study of *u*-RAFs will be performed in future work.

### Inhibition within a generalized RAF framework

Suppose we have a finite set *Y* and a partially order (finite or infinite) set *W*, together with some functions *f*:2^*Y*^→*W* and *g*:*Y*→*W*. Consider the fixed points of the map *ψ*:2^*Y*^→2^*Y*^ where *ψ*(*A*):={*y*∈*A*:*g*(*y*)≤*f*(*A*)}, other than *∅*. We are particularly interested in the setting where *f* is *monotonic* (i.e., where *A*⊆*B*⇒*f*(*A*)≤*f*(*B*)). We say that a subset *A* of *Y* is *gf-compatible* if *A* is non-empty and *ψ*(*A*)=*A*.

In [31], we showed that the RAFs in CRSs can be described by *gf*-compatibility. The fact that there is a polynomial time algorithm to find an RAF (if it exists, or else to determine that none exists) boils down to this ability to characterise RAFs by *gf*-compatibility, where *f* is monotonic and computable in polynomial-time, and the set of reactions and catalysts is finite. This is because the general problem of finding a *gf*-compatible set (if it exists) can be solved in general polynomial time when *Y* is finite and *f* monotonic. In [31], we showed how other problems (including a toy problem in economics) could be formulated within this more generalframework.

If we allow inhibition it is also possible to describe RAFs as *gf*-compatible sets, however the function *f* will generally not be monotonic. Briefly, we modify the construction as outlined in [31], where we work over the extended set of reactions (deleting any reaction that is not catalysed, and replacing any reaction that is catalysed by *k*>1 molecule types by *k* reactions where each catalyst is treated as a reactant of a corresponding reaction) by setting $Y= \mathcal {R}, W=2^{X}$ and, for *A*∈2^*Y*^, *f*(*A*)=cl_*A*_(*F*) and *g*(*r*)=*ρ*(*r*) (the ‘reactants’ of *r* (including a catalyst)). Note that in the definition of *f*(*A*), cl_*A*_(*F*) is the set of molecules present in *F* or constructible from *F* by a sequence of reaction from *A*, regardless of whether or not the catalyst for reactions are available. In that setting the RAFs in the CRS correspond to the *gf*-compatiblesubsets of .

Now, to allow inhibition, we simply replace *W*=2^*X*^ with *W*=2^*X*^×2^*X*^ (partially ordered by (*w*_1_,*w*_2_)≤(*w*1′,*w*2′)⇔*w*_1_⊆*w*1′ and *w*_2_⊆*w*2′) and replace *f* with *f*(*A*)=(cl_*A*_(*F*),*X*−cl_*A*_(*F*)), and *g*(*r*)=(*ρ*(*r*),in(*r*)), where in(*r*) denotes the subset of molecular species in *X* that inhibits reaction *r*. It follows that the *u*-RAFs in this CRS correspond to the *gf*-compatible subsetsof .

## Conclusions

The modified RAF algorithm, based on the notion of pseudo-RAFs, clearly improves on the number of calls to the closure computation procedure, which is computationally the most expensive part of the algorithm. Depending on the structure of catalysis in a reaction network, this can also lead to a significant improvement in the overall running time of the algorithm. However, in a purely “random” system (as in the binary polymer model), the additional overhead of keeping track of the number of reactions that produce a given molecule seems to cancel out the gain obtained from a reduced number of closure computations, at least for polymers of the sizes studied here. But, in general, the modified algorithm is never worse – and in some cases it is faster – than the original version.

Our statistical test on the expected number of irrRAFs within a reaction network provides strong support for theexistence of very large numbers of irrRAFs. In instances of the binary polymer model, even for *n*=8 with maxRAF sets of (on average) 375 reactions, is it highly likely (99% confidence) that at least *hundreds of millions* of irrRAFs exist. The overlap statistics show that these irrRAFs share, on average, about half of their reactions with each other, ranging from 25% to 80% overlap. In other words, there is always at least 20% (and up to 75%) difference between two arbitrary irrRAFs within the same reaction network. This large number and the relatively high variability could have important (and positive) consequences for the possible evolvability of autocatalytic sets [15,21].

Even though the general problem of finding RAF sets in systems that also allow inhibition (as well as catalysis) is NP-complete, we have shown that this problem becomes tractable when the total number of inhibitors is small (in absolute value). We have implemented and applied an extension of the basic RAF algorithm to search for such “uninhibited” RAF sets (*u*-RAFs), and have shown that their number and sizes depend largely on the level of inhibition (i.e., the probability that an inhibitor actually inhibits any given reaction) relative to the level of catalysis. So, at least in certain limited cases, inhibition can be dealt with efficiently. A full investigation of *u*-RAFs will be deferred to future work, however.

## Appendix

### pRAFs and HORN-SAT

Given a CRS with food set $Q = (X,\mathcal {R},C,F)$, we describe how the problem of finding the maximum pRAF subset of  can be expressed as an instance of the P-complete problem HORN-SAT (such a connection exists for any problem in the complexity class P [32], however finding an explicit description is often elusive). Recall that an instance of HORN-SAT consists of a set of literals, and a conjunction of *Horn clauses*. A Horn clause is a disjunction of literals involving at most one positive literal. The minimum truth assignment of literals which satisfies the proposition formula corresponds to the (unique) maximum pRAF subset of . We present here the construction of an instance of HORN-SAT from any given CRS.

First, it is necessary to construct the “expanded” CRS $\hat {Q} = (X, \hat {\mathcal {R}}, F)$ from the given CRS $Q = (X,\mathcal {R},C,F)$. The purpose of this expansion is to remove the consideration of catalysis when searching for RAFs or pRAFs in the system, thereby simplifying the process. This is achieved by considering catalysts as reactants of the reactions they catalyse. Formally, $\hat {\mathcal {R}}$ is obtained from  as follows: first delete every uncatalysed reaction. For each remaining $r \in \mathcal {R}$, let *c*(*r*) denote the set of distinct catalysts of *r*. Now replace *r* by |*c*(*r*)| reactions, each of which is identical to *r*, with the additions of one of the catalysts as a reactant (referred to as the nominated catalyst for that reaction). It was shown in [31] that RAFs in the original CRS correspond to RAFs in the expanded CRS; a similar argument shows that the expansion process also preserves the pRAF property.

Now consider the set of literals $Y \,=\, \left \{ y_{r} : r \in \hat {\mathcal {R}}\right \} \cup \left \{ y_{x} : x \in X\right \}.$, Given any subset $\mathcal {R}' \subseteq \hat {\mathcal {R}}$, let

$$y_{r} =\left\{ \begin{array}{ll} \texttt{true} & \quad\text{if } r \notin \mathcal{R}' \\ \texttt{false} & \quad\text{if } r \in \mathcal{R}' \end{array}\right. $$

for each $r \in \hat {\mathcal {R}}$, and let

$$y_{x} =\left\{ \begin{array}{ll} \texttt{true } & \quad\text{if } x \notin F\cup\pi(\mathcal{R}') \\ \texttt{false} &\quad \text{if } x \in F\cup\pi(\mathcal{R}') \end{array}\right. $$

for each *x*∈*X*.

We will construct a propositional Horn formula in conjunctive normal form, for which a truth assignment to literals in *Y* corresponds to a pRAF subset of $\hat {\mathcal {R}}$, and the minimal truth assignment corresponds to the maximum pRAF. First, note that the definition of a pRAF implies that for each reaction in $r \in \hat {\mathcal {R}}$, *r* is a member of some pRAF $\mathcal {R}' \subseteq \hat {\mathcal {R}}$ if and only if every reactant (including the nominated catalyst) is either a food molecule or is produced by some reaction in $\mathcal {R}'$ (this condition is both necessary and sufficient because we are working in the expanded CRS, so need not consider catalysis explicitly). This motivates the inclusion of the term

(2) $$\bigwedge_{r \in \hat{\mathcal{R}}} \left(\left(\bigwedge_{i=1}^{|\rho(r)|} \overline{y_{x_{i}}} \right) \vee y_{r} \right),  $$

where *x*_1_,…,*x*_|*ρ*(*r*)|_ are the reactants of *r*. The Horn formula must also relate the truth value of the literals {*y*_*x*_:*x*∈*X*} to membership of the set $F \cup \pi (\mathcal {R}')$. Motivated by this, we include the term

(3) $$ \bigwedge_{x \in X\setminus F} \left(\left(\bigvee_{i=1}^{k(x)} \overline{y_{r_{i}}}\right) \vee y_{x} \right),  $$

where *k*(*x*) is the number of distinct reactions producing *x*, and these reactions are *r*_1_,…,*r*_*k*(*x*)_. Satisfaction of this term requires that for each non-food molecule *x*, either some reaction producing *x* belongs to $\mathcal {R}'$, or the literal *y*_*x*_ is set to true. Finally, we include the term

(4) $$ \bigwedge_{x \in F} \overline{y_{x}},  $$

which simply requires that the truth value of every food molecule is set to false, since every food molecule trivially belongs to $F \cup \pi (\mathcal {R}')$.

Writing (2) and (3) in conjunctive normal form and combining with (4) gives the propositional Horn formula

(5) $$ \bigwedge_{x \in F} \overline{y_{x}}\bigwedge_{x \in X\setminus F} \left(\left(\bigvee_{i=1}^{k(x)} \overline{y_{r_{i}}} \right)\vee y_{x} \right)\bigwedge_{r \in \hat{\mathcal{R}}} \bigwedge_{i=1}^{|\rho(r)|}\left(\overline{y_{x_{i}}} \vee y_{r} \right)  $$

Any truth assignment satisfying (5) corresponds to a pRAF in $\hat {\mathcal {R}}$ (i.e. the set $\{r \in \hat {\mathcal {R}} : y_{r} = \texttt {false} \}$ is a pRAF), hence the unique minimal truth assignment satisfying the formula corresponds to the maximum pRAF in $\hat {\mathcal {R}}$.

### Pseudo-code for the modified RAF algorithm

**Variables:**

ᅟ *X* The set of molecule types.
ᅟ $\mathcal {R}'$ The current (sub)set of reactions.
ᅟ *x*, *p* A molecule type.
ᅟ r A reaction.
ᅟ *x*.clF A count of how many reactions in the current reaction set $\mathcal {R}'$ produce a molecule type *x* in the closure.
ᅟ *r*.clF Indicates whether a reaction *r* in the current reaction set $\mathcal {R}'$ is applied at least once during the computation of the closure.
ᅟ cl_*F*_ The closure of the food set as calculated so far.
ᅟ *ρ*(*r*) The reactants of a reaction *r*.
ᅟ *π*(*r*) The products of a reaction *r*.
ᅟ *γ*(*r*) The catalysts of a reaction *r*.
